# Editorial

**Published:** 1904-12-15

**Authors:** 


### EDITORIAL.

Social Standing of Dentists.—In another place we print an editorial from Dr. James Truman, on the social standing of the dentist, which expresses our sentiments exactly. Why a dentist, any more than a physician or a shoemaker, should want to associate with people with whom he can have no natural sympathies, is more than we can understand. We should feel as much out of place in an ultra-fashionable club as in a club of horsemen, or at a colored camp-meeting. But then we never belonged to a club and know nothing of the joys of living in such an atmosphere. It may be worth while striving for, but we know a hundred or more things we should rather be than a member of the swellest club in the world. Our nearest ambition is to be a first-class dentist.

Pacific Coast Dental Congress.—It is proposed to hold a Dental Congress in connection with the Lewis and Clark Exposition, which is to be held next summer in Portland, Oregon. The American Medical Association will hold its meeting there July 11th to 15th, and it is proposed to hold the Dental Congress the week before or the week after this meeting. Reduced railroad fares, as well as other inducements will make it a favorable time to visit this beautiful country.

Proceedings of Faculties Association for 1904.—The proceedings of the National Association of Faculties is at hand, containing the complete discussion of the question of the length of the course, including the Washington and St. Louis meetings. It is interesting reading and a very important document. The subject is not yet finally settled, and it will save a lot of needless discussion, to have this matter in shape for reference. It is a bit curious that the real reason for the change to the three year course comes out only once in the whole discussion. And when it is stated by one of the speakers in a straight-forward and manly way, it seems to catch the sympathy of every one, and really precipitates the deciding vote. Which only goes to prove that if the leaders of the faction desiring the change had come squarely out on this issue in the start, much acrimonious debate and the threatened disaster to the cause of education would have been saved. It was simply the fact that many colleges could not exist financially and do their work on the basis they wished, and which the professional advancement of the present time demands. This statement, put concretely into figures, was a clinching argument, and was instantly apprehended when made. The question now is, shall we advance standards at the expense of our educational institutions? It needs to be carefully considered before further action is taken.

---

THE COLLEGE OR A HIGHER STANDARD.—In the discussion before the National Association of Faculties, the dean of a prominent college made the statement that his school had not paid expenses the first year of the four-year course, and would lose several thousand dollars the coming year, unless the course required was changed. His school would either have to go out of business or curtail seriously its teaching capacity, at least twenty-five percent. It may be said that any school which could not maintain itself on the proper standard has no right to live. The school in question is one of the best, it is well equipped and ably managed, and it is unfortunate that it should be made to face such a crisis without ability to fall into line and wait for an increased attendance, which is sure to come later. The school in question is unfortunately located in the very midst of a large number of dental colleges, although the only one in its State. Within a radius of 275 miles are located nineteen other dental colleges, almost all well equipped and aggressively managed. No other college has over seventeen competitors in the same radius. The question is, shall this school, which has done excellent work be sacrificed for the sake of a higher educational standard? Some of its competitors could be spared with no serious loss, but who shall say, which ones! The subject will need to be carefully considered before another radical step is taken.

